# Testing Invariance of Measures of Internalizing Symptoms Before and After a Major Life Stressor: The Impact of COVID-19 in an Adolescent and Young Adult Sample

**DOI:** 10.1177/10731911211015315

**Published:** 2022-10

**Authors:** Thomas M. Olino, Julia A. C. Case, Mariah T. Hawes, Aline Szenczy, Brady Nelson, Daniel N. Klein

**Affiliations:** 1 Temple University, Philadelphia, PA, USA; 2 Stony Brook University, Stony Brook, NY, USA

**Keywords:** psychometrics, COVID-19, measurement invariance, depression, anxiety, assessment

## Abstract

There are reports of increases in levels of internalizing psychopathology during the COVID-19 pandemic. However, these studies presume that measurement properties of these constructs remained unchanged from before the pandemic. In this study, we examined longitudinal measurement invariance of assessments of depression, anxiety, and intolerance of uncertainty (IU) in adolescents and young adults from ongoing longitudinal studies. We found consistent support for configural and metric invariance across all constructs, but scalar invariance was unsupported for depression and IU. Thus, it is necessary to interpret pandemic-associated mean-level changes in depression and IU cautiously. In contrast, mean-level comparisons of panic, generalized, and social anxiety symptoms were not compromised. These findings are limited to the specific measures examined and the developmental period of the sample. We acknowledge that there is tremendous distress accompanying disruptions due to the COVID-19 outbreak. However, for some instruments, comparisons of symptom levels before and during the pandemic may be limited.

Numerous reports have concluded that during the spring and summer of 2020, the COVID-19 global pandemic led to increased levels of psychological distress (Loades et al., 2020; Vindegaard & Benros, 2020). This increase can be attributed both to the direct effects of the virus, and to the effects of imposing stringent public health measures to curb the pandemic, such as social distancing, quarantines, and stay at home orders. Determining whether changes in the level of distress are due to COVID-19 is contingent on the instruments having the same measurement properties before and into the pandemic. Thus, this study examines multiple levels of measurement invariance (MI; Millsap, 2011; Widaman et al., 2010) in measures of internalizing psychopathology, including depression and anxiety, and a correlate of these constructs, intolerance of uncertainty (IU), across adolescents and young adults participating in two longitudinal studies of youth development that preceded the COVID-19 outbreak. The results may have important implications for understanding reports of changes in symptoms during this period.

When examining change in psychological constructs, it is critical to show that the psychometric assessments of those constructs are consistent across time. This property is termed “MI” (Millsap, 2011; Widaman et al., 2010). When MI is supported, as evinced by configural (i.e., common presence of indicators), metric (i.e., noninvariance of factor loadings), and scalar (i.e., noninvariance of intercepts) invariance, mean-level comparisons of the target construct, as assessed by a particular instrument, are valid. Support for metric invariance would indicate that examination of associations with respect to rank-order (e.g., correlation, regression) would be valid. Furthermore, support for scalar invariance would indicate that examination of mean-level differences (e.g., between groups; across time; across contexts) are valid. There may also be mixed-support with only a subset of items demonstrating metric and scalar invariance. This is termed partial metric and partial scalar invariance.

Longitudinal studies of MI have primarily used naturalistic observational designs in youth and early adulthood. For example, Mathyssek et al. (2013) examined MI of youth self-reports of depression and anxiety in a large (*N* = 2,230) epidemiologic study spanning aged 11 to 16 years using the Revised Child Anxiety and Depression Scale (Chorpita et al., 2000). The authors found strong support (i.e., trivial reductions in model fit) for metric invariance, but modest support (i.e., some aspects of fit showed substantive reduction in fit) for scalar invariance. However, models continued to be an excellent fit to the data. Based on this evidence the authors concluded that the comparisons of constructs are valid across this developmental span. Olino et al. (2018) examined longitudinal metric and scalar MI of anxiety in a moderately sized (*n* = 487) sample of youth from age 9 to 12. For symptoms of generalized anxiety disorder (GAD), panic disorder, and social anxiety disorder using the Screen for Child Anxiety Related Emotional Disorders (SCARED; Birmaher et al., 1997), there was sufficient metric and scalar MI to support comparisons of levels of these symptom dimensions over time. Wu (2017) examined MI of the Beck Depression Inventory in a study of adolescents (*n* = 730) spanning ages 13 to 16 years. The author found support for configural, metric, and scalar invariance of a three-dimensional model (negative attitudes, performance difficulties, and somatic complaints). Similarly, Leadbeater et al. (2012) found support for configural and metric, but not scalar invariance of depressive and anxiety symptoms across four waves of data collection (*n* = 662) spanning ages 12 to 26 years using the Brief Child and Family Phone Interview (Cunningham et al., 2009). Finally, Tyrell et al. (2019) found configural, metric, and scalar invariance of two measures of depressive symptomatology, an abbreviated version of the Children’s Depression Inventory (CDI) and the Anxious/Depressed subscale of the Adult Self-Report (ASR) from ages 12 to 21 years (*n* = 392). Thus, these studies show general support, with some exceptions, for MI over the course of adolescence and early adulthood.

There have been fewer examinations of MI when participants experience contextual changes. Fried et al. (2016) examined longitudinal MI in adults (*n* = 649) as they completed interventions for depression. The authors found that the structure of the depression measures—the Hamilton Rating Scale for Depression, the clinician-rated Inventory of Depressive Symptoms, the clinician-rated Quick Inventory of Depressive Symptoms, and the self-rated Inventory of depressive symptoms—changed in such a fashion, including the number of factors needed to explain the observed data, that comparisons across time were not valid. There also have been several studies (Goodman-Williams & Ullman, 2020; Lommen et al., 2014; Platt et al., 2016; Wang et al., 2012) that have examined MI across multiple assessments following natural disasters or unpredictable stressors. However, these studies are not able to speak to changes in psychometric functioning from before to after these exposures. Other studies have examined MI across predictable, but stressful traumatic events, such as before and after deployment into military conflict contexts (Contractor et al., 2017). Contractor et al. (2017) failed to find support for MI (i.e., factor loadings and/or item thresholds were not equivalent) for the measurement of symptoms of posttraumatic stress disorder before and after deployment (*n* = 867). In a different study, Miller et al. (2018) examined MI in a measure of sleep problems in spouses of military members (*n* = 686) before and during periods of deployment. The authors found support for invariance, permitting rank-order and mean-level comparisons across assessments. Thus, exposure to some contexts appears to influence the measurement of some constructs in some individuals, but effects may vary depending on the event, measure, and sample examined. However, studies examining MI before and after significant life events have not yet been conducted spanning adolescence and early adulthood.

Beyond examination of symptoms, there are multiple correlates of internalizing problems that may also be of particular interest. One such construct is IUS (Carleton, 2016a, 2016b; Freeston et al., 1994). We included this as a construct of interest in the current study, as diffuse fears of infection and safety were heightened during the initial outbreak (Center for Disease Control and Prevention, 2020). We are aware of only one study examining MI in the context of Intolerance of Uncertainty Scale (IUS). This study found evidence for partial invariance across sex in adolescents (Dekkers et al., 2017).

The previous work examining longitudinal changes in the context of major life stressors relied on events with temporally well-defined onsets and offsets. In contrast, during the COVID-19 pandemic, potential exposure to the virus is ongoing and uncertain. Moreover, public health interventions, particularly stay at home orders and quarantines, may have impacts of their own. Thus, the impact of the pandemic may have stronger or more persistent impacts on functioning than events with demarcated onsets and offsets. Here, we examine MI from the assessment of symptoms of depression and anxiety and the construct of IU (Buhr & Dugas, 2002) in a sample composed of participants (*n* = 505) from two ongoing studies of youth on Long Island, NY, one of the initial epicenters of the pandemic (Hawes et al., 2021). Initial measures were completed during planned study assessments and follow-up assessments were completed between March 27th and May 15th 2020, spanning roughly the 1.5 months following the New York state shut-down order (Jacobs et al., 2020). Although there is mixed evidence for specific events (e.g., military deployment; psychological interventions) affecting measurement properties of symptom scales, we hypothesized that the experience of the COVID-19 pandemic would be associated with altered measurement properties of depression, anxiety, and IU.

## Method

### Participants

#### Stony Brook Temperament Study (SBTS)

The SBTS is an ongoing longitudinal study designed to explore early antecedents and pathways to depressive and anxiety disorders in a community-based sample of youth from Long Island, NY (Klein & Finsaas, 2017). Families with a 3-year-old child were contacted through commercial mailing lists and were eligible to participate if the primary caretaker spoke English and was the child’s biological parent, and if the child did not have a significant medical disorder or developmental disability. One child per family was enrolled. Children and their families were invited to participate in follow-up assessments every 3 years. Pre-COVID measures were taken from the 5th wave of data collection. This was the nearest assessment to the start of the pandemic and the wave with the availability of the same measures administered during the pandemic. SBTS participants were between the ages of 14 and 17 years (*M* = 15.01, *SD* = 0.37, *n* = 308) at the pre-COVID wave and 15 and 19 years (*M* = 17.09, *SD* = 0.86, *N* = 335) during COVID survey. The SBTS sample reported on here is predominantly female (52.8%), White/non-Hispanic (91.6%), with college-educated mothers (57.4%). Between 9 months and 4 years elapsed between participants’ pre-COVID and during COVID assessments (*M* = 2.05 years, *SD* = 0.86). Self-reports were administered in-person for the pre-COVID and remotely for the during COVID assessments.

#### Impact of Puberty on Affect and Neural Development Across Adolescence (iPANDA) Project

The iPANDA project is an ongoing, multimethod, longitudinal study aimed at investigating within-subject trajectories of reward sensitivity and depressive symptoms in a large community sample of adolescent girls from Long Island, NY (Burani et al., 2019). Participants were recruited using a commercial mailing list of families in the Stony Brook area with daughters between the ages of 8 and 14 years, as well as through flyers, online postings, and references from participating families. Families who met eligibility criteria, which included having a daughter in the targeted age range with no known medical or developmental disability, a biological parent willing to participate, and ability to read and write English, were invited to participate in the study. Following the first wave of data collection, the girls were reassessed at 2-year time intervals through adolescence. Pre-COVID measures were taken from the 2nd wave of data collection. This was the nearest assessment to the start of the pandemic and the wave with the availability of the same measures administered during the pandemic. Participants from the iPANDA project were between the ages of 10 and 17 years (*M* = 14.13, *SD* = 1.72, *n* = 160) at the pre-COVID wave and 12 and 22 years (*M* = 18.38, *SD* = 1.82, *n* = 170) at the during COVID survey. The sample is predominantly White/non-Hispanic (82.9%), with a college-educated parent (72.4%). Between 2 and 6 years elapsed between participants’ pre-COVID and during COVID assessments (*M* = 4.19 years, *SD* = 0.86). Self-reports were administered in-person for the pre-COVID and remotely for the during COVID assessments.

### Procedures

In both samples and at both pre-COVID and during COVID assessments, consent was obtained by parents of participants younger than 18 years prior to contacting the minor directly. After obtaining written informed consent from individuals 18-years-old or older, or assent from individuals younger than 18 years, participants completed a battery of questionnaires, including measures of depression, anxiety, and IU. Study procedures were approved by the Stony Brook University Institutional Review Board.

### Measures

#### Children’s Depression Inventory

The CDI is a 27-item self-report questionnaire designed to assess symptoms of depression occurring in the past 2 weeks in youth aged 7 to 17 years (Kovacs, 1984). Items are rated on a 3-point scale, ranging from *symptom absent* (0) to *symptom present* (2), and summed to create a total severity of depressive symptoms score. Adequate reliability and validity of the CDI in assessing depression in youth has been demonstrated (Dougherty et al., 2018). In our sample, the CDI possessed excellent internal consistency (α = .90).

#### Screen for Child Anxiety Related Emotional Disorders

The SCARED is a 41-item inventory intended to measure anxiety disorder symptoms over the past month in youth aged 8 to 18 years (Birmaher et al., 1997). The SCARED is composed of 5 subscales capturing different clusters of anxiety symptoms (somatic/panic, general anxiety, separation anxiety, social phobia, and school phobia) that can be summed to create a total anxiety score. Participants rate the frequency of symptoms from *almost never* (0) to *often* (2). The school phobia subscale was not administered at the during COVID assessment, so the current analyses are based on the remaining 37 items. The SCARED has demonstrated good psychometric properties in youth samples (Rappaport et al., 2017). Internal consistency was excellent in our sample (α *=* .94).

#### Intolerance of Uncertainty Scale

The IUS-12 is a short form of the original 27-item IUS. It contains 12 items that are designed to assess emotional, cognitive, and behavioral responses to uncertainty as well as implications of being uncertain and attempts to control the future (Carleton et al., 2007). Participants rate items on a 5-point Likert-type scale, ranging from *not at all characteristic of me* (1) to *entirely characteristic of me* (5). Items can be summed to create a total score. The IUS-12 has been shown to be a reliable and valid measure of IU in clinical and nonclinical samples (Khawaja & Yu, 2010). In the current sample, internal consistency for the IUS total score was excellent (α = .92). The IUS-12 has been used extensively as a single factor measure, but has also been examined having a two-factor structure with prospective and inhibitory factors.

### Statistical Analyses

In line with a model building approach and to identify whether one-factor models were appropriate for testing, we estimated a series of initial single-factor CFAs separately for youth self-reports of depression, anxiety, and IUS at the longitudinal assessment preceding and shortly after the peak of the COVID-19 pandemic in New York. These models are foundational before testing MI. We examined unidimensional models for each measure. If measures did not show at least adequate fit, we examined multidimensional structures. If multidimensional models did not yield adequate fit, we examined model residuals to identify areas of model strain. When indicated, model modifications were made to enhance model fit. Next, models were fit sequentially to evaluate MI. We followed the progression of testing MI used in examinations of longitudinal invariance (Widaman et al., 2010). In all longitudinal models, we included residual covariances between the same items across time. We tested first for configural invariance, or whether the pattern of significant (i.e., non-zero) factor loadings was similar across assessment waves while permitting the factors to be correlated. These models were specified freely estimating all factor loadings and thresholds and fixing the latent variable variance at 1 for purposes of model identification. Next, we tested for metric invariance, or whether factor loadings for each item were equal across assessments. In these models, we freely estimated the variance of the latent factor at the COVID-19 assessment as fixing factor loadings to be equal across time permits this constraint to be relaxed for one assessment wave. Finally, we tested for scalar invariance, or whether the probability of item endorsement was similar across time, by constraining the thresholds across waves to be equal. In these models, we freely estimated the mean of the latent factor at the COVID-19 assessment, as fixing thresholds to be equal across assessments permits this constraint to be relaxed for one occasion. If all three types of invariance hold, this indicates that the scales measure the same constructs across time on the same scale. Thus, differences in mean trait levels can be interpreted as true score differences, as opposed to differences in measurement.

For models that did not achieve full MI, we tested partial MI, to identify whether some, but not all, items were invariant across informants and/or time. We examined differences in factor loadings using the MODEL CONSTRAINT command in M*plus* to assess differences in configural invariance. When factor loadings were identified that did not significantly differ at *p* < .05, a partial metric invariant model was estimated that included equality constraints on those factor loadings, while allowing other factor loadings to be freely estimated. In this partial metric invariance model, we also used the MODEL CONSTRAINT command to test whether the between threshold parameters significantly differed, to examine the presence of comparable item thresholds. When item thresholds were identified that did not significantly differ at *p* < .05, a partial scalar invariant model was estimated that included equality constraints on those item thresholds, while allowing other thresholds to be freely estimated. The supplementary materials (https://osf.io/hpxa5/) provide complete information for these model tests.

All models were estimated in M*plus* version 8 (Muthén & Muthén, 1998) using the weighted least squares estimator (Flora & Curran, 2004), which is a robust estimator suited for modeling ordinal data. We evaluated models on two goodness of fit indices. Specifically, we used the comparative fit index (CFI; Bentler, 1990) and root mean square error of approximation (RMSEA; Steiger, 1990). Although cut-offs are somewhat arbitrary (Marsh et al., 2004), current conventions suggest that excellent model fit is indicated by CFI values ≥.95 (Hu & Bentler, 1999) and RMSEA values ≤.05 (MacCallum et al., 2006); and adequate fit is indicated by CFI greater than .90 and a RMSEA between .05 and .08. Model fit comparisons were evaluated by investigating change in both CFI and RMSEA using Chen’s (2007) guidelines across levels of invariance (i.e., configural vs. metric; metric vs. scalar). Chen (2007) recommended interpreting reductions in CFI of .01 and RMSEA of .015 as indicating noninvariance (i.e., failure to demonstrate MI). Implementation of the models was supported by the *MplusAutomation* package (Hallquist & Wiley, 2018) in R (R Core Team, 2018).

## Results

### Examination of Unidimensional Models

We first fit unifactorial models to the items comprising each of the measures (Table 1). For the CDI and IUS pre- and during COVID-19 assessments, there was at least adequate fit for the individual models, although the pre-COVID-19 IUS model had a RMSEA value higher than would be preferred. There was also equivocal fit for a two-factor model of the IUS with correlated prospective and inhibitory IU dimensions. Inclusion of up to two post hoc residual covariance paths did not substantially improve fit. Thus, we proceeded with the unidimensional model for the IUS. Substantively similar conclusions were reached when analyses were repeated on the two-factor IUS model.

**Table 1.** Overall Fit for Pre- and During COVID-19 Pandemic.

	χ^2^	*df*	CFI	RMSEA
CDI				
Pre-COVID	631.705	324	0.959	0.036 (0.032-0.041)
During COVID	714.004	324	0.949	0.049 (0.044-0.054)
IUS				
Pre-COVID	473.147	54	0.916	0.106 (0.097-0.114)
During COVID	243.370	54	0.977	0.084 (0.074-0.095)
SCARED				
During COVID one-factor model	3507.516	629	0.831	0.096 (0.093-0.099)
Pre-COVID one-factor model	3255.302	629	0.848	0.076 (0.074-0.079)
Pre-COVID four factor model	1775.322	623	0.933	0.051 (0.048-0.054)
During COVID four factor model	3024.376	623	0.859	0.088 (0.085-0.091)
SCARED panic				
Pre-COVID	184.856	65	0.972	0.051 (0.042-0.060)
During COVID	157.954	65	0.980	0.054 (0.043-0.065)
SCARED GAD				
Pre-COVID	248.870	27	0.961	0.107 (0.095-0.120)
During COVID	233.827	27	0.958	0.125 (0.110-0.139)
Pre-COVID GAD modification	168.334	26	0.975	0.088 (0.075-0.100)
During COVID GAD modification	144.809	26	0.976	0.096 (0.081-0.112)
SCARED separation				
Pre-COVID	185.099	20	0.899	0.108 (0.094-0.122)
During COVID	151.741	20	0.846	0.115 (0.099-0.133)
Pre-COVID separation modification	80.384	18	0.962	0.070 (0.055-0.086)
During COVID separation modification	59.692	18	0.951	0.068 (0.050-0.088)
SCARED social				
Pre-COVID	53.956	14	0.992	0.063 (0.046-0.081)
During COVID	105.422	14	0.980	0.115 (0.095-0.136)
Pre-COVID social modification	44.698	13	0.993	0.058 (0.040-0.078)
During COVID social modification	70.681	13	0.987	0.095 (0.074-0.117)

*Note*. CDI = Child Depression Inventory; IUS = Intolerance of Uncertainty Scale; SCARED = Screen for Childhood Anxiety and Related Disorders; GAD = generalized anxiety disorder symptoms; Panic = panic disorder symptoms; and Social Anxiety = social anxiety symptoms; *df* = degrees of freedom; CFI = comparative fit index; RMSEA = root mean square error of approximation.

For the SCARED, we first tested a unidimensional model. This was a poor fit to the data for both the pre- and during COVID-19 assessments. We then tested a correlated four-factor model. Fit for the pre-COVID-19 assessment model was good, but the model was a poor fit to the data for the during COVID-19 assessment. Thus, we proceeded to test the individual anxiety subscales of the SCARED separately. The fit for the pre- and during COVID-19 assessments of panic symptoms was very good. For the GAD, separation anxiety, and social anxiety scales, the initial unidimensional models did not consistently fit the data well. We added one post hoc residual covariance path in the GAD (Items 23 and 28) and social anxiety (Items 3 and 10) models and two post hoc residual covariance paths in the separation anxiety models (Items 16 and 20 and Items 8 and 29). After including these residual covariance paths, the models fit the data at least adequately.

### Tests of Measurement Invariance

Results of tests of longitudinal MI are shown in Table 2. For the CDI, the unidimensional model of longitudinal configural invariance was a good fit to the data. Introduction of equality constraints to the factor loadings to like items across assessments did not show a decrement in model fit. However, when applying constraints on the thresholds for testing scalar invariance, the model showed a substantial reduction of the CFI and RMSEA. Direct tests of equivalence of like thresholds showed that 48 of 54 thresholds significantly differed across time. Only 4 of 27 items had noninvariant thresholds across time. Estimating a partial scalar invariant model for which item thresholds for those four items were constrained to equality across time showed a good fit to the data. When there were significant differences in item thresholds across time, comparisons showed that the thresholds for pre-COVID assessments were higher than those during the COVID pandemic.

**Table 2.** Tests of Longitudinal Measurement Invariance.

	χ^2^	*df*	CFI	RMSEA	Comparison	ΔCFI	ΔRMSEA
CDI							
1. Configural	2069.082	1349	0.946	0.027 (0.024-0.029)			
2. Metric	2069.384	1375	0.948	0.026 (0.024-0.028)	2 vs. 1	0.002	−0.001
3. Scalar	3800.914	1428	0.823	0.047 (0.045-0.049)	3 vs. 2	−0.125	0.021
4. Partial scalar	2074.147	1382	0.948	0.026 (0.023-0.028)	4 vs. 2	0.000	0.000
IUS							
1. Configural	594.946	239	0.969	0.045 (0.04-0.049)			
2. Metric	658.517	250	0.964	0.047 (0.042-0.051)	2 vs. 1	−0.005	0.002
3. Scalar	1395.033	297	0.904	0.07 (0.067-0.074)	3 vs. 2	−0.060	0.023
4. Partial scalar	667.703	257	0.964	0.046 (0.042-0.051)	4 vs. 2	0.060	−0.024
SCARED panic							
1. Configural	466.566	285	0.977	0.029 (0.024-0.034)			
2. Metric	462.445	297	0.979	0.027 (0.022-0.032)	2 vs. 1	0.002	0.002
3. Scalar	569.481	322	0.969	0.032 (0.028-0.036)	3 vs. 2	−0.010	0.005
SCARED GAD							
1. Configural	331.330	123	0.978	0.047 (0.041-0.054)			
2. Metric	306.202	131	0.982	0.042 (0.036-0.048)	2 vs. 1	0.004	0.005
3. Scalar	392.151	148	0.974	0.047 (0.041-0.052)	3 vs. 2	−0.008	0.005
SCARED separation							
1. Configural	246.395	91	0.941	0.048 (0.041-0.055)			
2. Metric	242.971	98	0.945	0.044 (0.037-0.051)	2 vs. 1	0.004	0.004
3. Scalar	308.181	113	0.926	0.048 (0.042-0.054)	3 vs. 2	−0.019	0.004
4. Partial scalar	247.695	103	0.945	0.043 (0.036-0.050)	4 vs. 2	0.000	−0.001
SCARED social							
1. Configural	144.252	67	0.992	0.039 (0.030-0.048)			
2. Metric	242.134	73	0.981	0.055 (0.048-0.063)	2 vs. 1	−0.011	0.016
3. Partial metric	136.234	71	0.993	0.035 (0.026-0.044)	3 vs. 1	0.001	−0.004
4. Scalar	150.623	78	0.992	0.035 (0.027-0.044)	4 vs. 3	−0.001	0.000

*Note*. Changes in CFI and RMSEA are calculated as differences between the noted models in the comparison column. CDI = Child Depression Inventory; IUS = Intolerance of Uncertainty Scale; SCARED = Screen for Childhood Anxiety and related Disorders; GAD = generalized anxiety disorder symptoms; Panic = panic disorder symptoms; and Social Anxiety = Social Anxiety Symptoms; *df* = degrees of freedom; CFI = comparative fit index; RMSEA = root mean square error of approximation.

For the Panic/Somatic and GAD SCARED subscales, longitudinal configural invariance models were both a good fit to the data. Introduction of equality constraints to the factor loadings to like items across assessments did not show a decrement in model fit. Similarly, when applying equality constraints on the item thresholds, there was not a significant decrement in model fit for either dimension. Thus, there was support for full scalar invariance for these subscales.

For the separation anxiety SCARED subscale, the configural invariance model was a good fit to the data. Introduction of equality constraints to the factor loadings to like items across assessments did not show a decrement in model fit. However, when applying constraints on the thresholds for testing scalar invariance, the model showed a substantial reduction of the CFI and RMSEA. Direct tests of equivalence of like thresholds showed that 6 of 16 thresholds significantly differed across time. Only three of eight items had noninvariant thresholds across time. Estimating a partial scalar invariant model for which item thresholds for those three items were constrained to equality across time also showed a good fit to the data.

For the Social Anxiety SCARED subscale, the configural invariance model was a good fit to the data. Introduction of equality constraints to the factor loadings to like items across assessments led to a decrement in model fit according to the RMSEA. Direct tests of equivalence of like factor loadings showed that two of seven loadings significantly differed across time. The partial metric invariance model was a good fit to the data. Introduction of equality constraints to the thresholds of like items across assessments did not show a decrement in model fit. Because of noninvariance of two-factor loadings, thresholds for those items were not constrained to be equal in this model. Thus, six of eight total items had factor loadings and thresholds constrained to be equal for this subscale and partial scalar invariance was supported for Social Anxiety.

For the IUS, the unidimensional model of longitudinal configural invariance was a good fit to the data. Introduction of equality constraints to the factor loadings to like items across assessments did not show a decrement in model fit. However, when applying constraints on the thresholds for testing scalar invariance, the model showed a substantial reduction of the CFI and RMSEA. Direct tests of equivalence of like thresholds showed that 31 of 48 thresholds significantly differed across time. Only 2 of 12 items had noninvariant thresholds across time. When there were significant differences in item thresholds across time, comparisons showed that 19 of the 31 thresholds for pre-COVID assessments were lower than those during the COVID pandemic. Estimating a partial scalar invariant model for which item thresholds for those two items were constrained to equality across time also showed a good fit to the data. Analyses were estimated for a correlated prospective and inhibitory two-factor model. Overall, the same pattern of findings was obtained, with supportive evidence for configural and metric invariance, but a lack of support for scalar invariance.

## Discussion

There have been extensive concerns and multiple reports of increased internalizing symptomatology during the COVID-19 global pandemic (Loades et al., 2020; Vindegaard & Benros, 2020). In evaluating the magnitude of changes in levels of depression, anxiety, and other relevant and closely related constructs, such as IU, it is critical to identify whether the meaning of the constructs is the same during the pandemic as it was before this period. This requires evaluation of MI (Millsap, 2011; Widaman et al., 2010). Previous psychometric studies examining changes in the psychometric functioning of measures as a function of exposure to natural disasters have been limited as assessments were only available after the event (Goodman-Williams & Ullman, 2020; Lommen et al., 2014; Platt et al., 2016; Wang et al., 2012). Some previous research, such as studies of military deployment (Contractor et al., 2017; Miller et al., 2018), have had both pre- and post-event data. However, the results of those studies have been mixed. The pandemic is a somewhat unique stressor, as its time course is less clearly delimited. Nonetheless, a critical challenge in evaluating the effects of the pandemic on mental health is the availability of psychometrically equivalent assessments both before and during the pandemic. In this study, we capitalized on data from two ongoing longitudinal studies of adolescents and young adults who completed measures of depression, anxiety, and IU several years prior to the pandemic, and around the time of its peak in the New York area. There was support for configural and metric invariance that suggests that the results of research examining the relative stability of symptoms (e.g., multiple linear regression analyses) can be interpreted with confidence from a measurement perspective. However, we found mixed support for scalar invariance. This suggests caution in interpreting mean-level increases in symptoms from before to during the COVID-19 pandemic.

For the SCARED Panic/Somatic and GAD subscales, we found support for scalar invariance. Our results provide strong support for the psychometric validity of tests of mean-level change from pre- to during the COVID-19 pandemic. This indicates that changes in construct scores are not contaminated by differences in measurement properties of the items.

The models for the Social Anxiety subscale also showed support for consistent measurement across time. For this model, however, there were two items (“I feel shy with people I don’t know well.” and “I am shy”) that showed different relationships with the underlying latent factor. The changes in loadings for these items were not systematic, with one item having an increase and the other a decrease in magnitude across time. However, aside from these items, further constraints did not substantially worsen model fit. Thus, there was good evidence for comparability of social anxiety across assessments.

For the separation anxiety subscale, we observed only partial scalar invariance. The partial scalar invariance model had three items for which all item thresholds did not significantly differ across time. Previous work (Hambleton et al., 1991; Kolen & Brennan, 2014; Pokropek et al., 2019) has suggested that factors with only a few items showing invariance of thresholds are robust enough to anchor mean-level comparisons. However, mean-level comparisons may be more tenuous, as few items showed equivalence across assessments.

For the assessment of depression, the loadings of the items were consistent over time. However, the model substantially deteriorated when applying constraints on the item thresholds, indicating that there were differences in these loadings across time. The thresholds for endorsing higher levels of depression were typically lower during, relative to before, the COVID-19 pandemic. This indicates that, given a similar level of depression, participants were more likely to endorse more severe response options during the pandemic. We speculate that media attention to the impact of social distancing on experience of isolation and depressive symptoms may have led reduced stigma surrounding reporting of symptoms. This may have manifest in the lowered thresholds of endorsement of those items, in particular. Thus, the evidence suggests that there are important changes in the measurement properties of depression assessments, complicating the interpretation of mean-level changes in the CDI as a function of the pandemic.

The conclusions regarding IU are mixed. Similar to depressive symptoms, we failed to find support for scalar invariance, as only two items had thresholds that did not differ across time. Thus, there is not adequate information with which to calibrate scaling across time. In contrast to the results for depression, however, the pattern of changes in item thresholds was not consistent. Over half of the intercepts were lower pre- than during the COVID pandemic. This suggests that, given the same levels of latent IUS, participants were less likely to endorse higher severity responses for some items, but more likely to endorse higher severity options for other items, during the COVID pandemic.

In sum, there is mixed evidence for the equivalence of some psychometric characteristics of measures of depression, anxiety, and IU as adolescents and young adults live through a pandemic. This basic measurement work suggests that it can be challenging to evaluate some of the results from the burgeoning research on mental health outcomes of COVID-19, and potentially for studies of the effects of disasters, traumas, and life stressors on psychological functioning. Our results suggest that although relative levels of symptoms appear to be rated consistently from before to during the pandemic (i.e., as metric invariance was found for nearly all measures/scales), ratings of the absolute levels of some symptoms may not be comparable. Thus, interpreting analyses examining absolute change (i.e., changes in levels) of symptoms requires careful attention as these patterns appear to vary as a function of constructs or measures, and perhaps due to the nature of the event and the sample.

This work benefits from two ongoing longitudinal studies for which assessments using the same instruments were obtained from the same participants before and during the pandemic. Thus, our analyses are sensitive in that we have a fully within-person design. A key strength to emphasize is the locale and timing of the study. Long Island was at one time the world’s epicenter of the pandemic, and we were able to capture data in this location and period in this study (Jacobs et al., 2020).

There also are important limitations to consider. Psychometric functioning for these scales may have differed because of developmental processes, rather than because of changes due to stressful life experiences during the COVID-19 pandemic. However, the large range of ages of respondents and overlap of age ranges in the before to during COVID assessments somewhat mitigates this concern. For anxiety and depression, previous work has supported longitudinal MI (Mathyssek et al., 2013; Olino et al., 2018; Stumper et al., 2019). Stumper et al. (2019) used the CDI and Olino et al. (2018) used the SCARED. Thus, there is some support that these measures show longitudinal invariance. However, these data come from naturalistic studies of repeated measures without the experience of a shared stressor, whereas the present study focused on change as a function of a pandemic. Unfortunately, there are no published studies of longitudinal MI for the IUS. Thus, we cannot leverage previous studies to discern whether our IUS results may be confounded by development. The pre- and during COVID assessments also differed in administration method. Thus, we cannot rule out that this assessment procedural difference may have affected results. However, a systematic review of studies examining comparability of internet and in-person administration (Alfonsson et al., 2014) concluded minimal impact of the effects of these changes. The results of this work are also limited to the specific measures examined and the models tested. Alternative models, such as network models, may show different patterns of changes across waves (e.g., Curtiss et al., 2018).

Based on these results, we find that comparisons of depression, IUS, and some, but not all, dimensions of anxiety are not entirely psychometrically equivalent from before to during the COVID-19 pandemic. Thus, caution is required in interpreting comparisons of levels of internalizing problems as a function of COVID-19. While mean-level change of some measures may be difficult to interpret, other indices, such as relative change (i.e., rank-order), should be largely unbiased. These measurement concerns, however, do not take away from individuals’ experience of distress during this time. It will be critical to evaluate whether the psychometric functioning of these measures changes further as the pandemic wanes.
